# The anti-arthritic, anti-inflammatory, antioxidant activity and relationships with total phenolics and total flavonoids of nine South African plants used traditionally to treat arthritis

**DOI:** 10.1186/s12906-016-1301-z

**Published:** 2016-08-23

**Authors:** Ishaku Leo Elisha, Jean-Paul Dzoyem, Lyndy Joy McGaw, Francien S. Botha, Jacobus Nicolaas Eloff

**Affiliations:** 1 Phytomedicine Programme, Department of Paraclinical Sciences, Faculty of Veterinary Science, University of Pretoria, Private Bag X04, Onderstepoort, 0110 Pretoria, South Africa; 2 Permanent address: Drug Development Section, Biochemistry Division, National Veterinary Research Institute, P.M.B 01, Vom-Plateau State, Nigeria; 3 Permanent address: Department of Biochemistry, Faculty of Science, University of Dschang, P.O. Box 67, Dschang, Cameroon

**Keywords:** Medicinal plants, Anti-lipoxygenase, Anti-arthritic activity, Inhibition of nitric oxide production, Antioxidant activity, Phytochemicals

## Abstract

**Background:**

Oxidative stress predisposes the human and animal body to diseases like cancer, diabetes, arthritis, rheumatoid arthritis, atherosclerosis and chronic inflammatory disorders. Hence, this study seeks to determine the antioxidant, anti-inflammatory and anti-arthritic activities of acetone leaf extracts of nine South African medicinal plants that have been used traditionally to treat arthritis and inflammation.

**Methods:**

The anti-inflammatory activity of the extracts was determined by investigating inhibition of nitric oxide production in lipopolysaccharide activated RAW 264.7 macrophages as well as 15-lipoxygenase enzyme inhibition. An anti-protein denaturation assay was used to determine the anti-arthritic properties of the extracts. The antioxidant activity was determined using the 2,2-diphenyl-1-picrylhydrazyl (DPPH), 2,2′-azino-bis (3-ethyl-benzthiazoline-6-sulfonic acid) (ABTS) radical scavenging assays and ferric reducing antioxidant power (FRAP). The total phenolic and total flavonoid concentration of extracts were determined by using standard methods.

**Results:**

All extracts inhibited nitric oxide production in a dose-dependent manner in the LPS-stimulated RAW 264.7 macrophages. Extracts of *Maesa lanceolata* and *Heteromorpha arborescens* inhibited NO production by 99.16 % and 89.48 % at a concentration of 30 μg/ml respectively. *Elaeodendron croceum* and *Calpurnia aurea* extracts had strong activity against 15-lipoxygenase activity with IC_50_ values of 26.23 and 34.70 μg/ml respectively. *Morus mesozygia* and *Heteromorpha arborescens* extracts had good in vitro anti-arthritic activity with IC_50_ values of 11.89 and 53.78 μg/ml, the positive control diclofenac sodium had IC_50_ value of 32.37 μg/ml. The free radical scavenging activity of the extracts in DPPH assays ranged between 7.72 and 154.77 μg/ml. Trolox equivalent antioxidant capacity (TEAC) and FRAP values ranged from 0.06 to 1.32 and 0.06 to 0.99 respectively.

**Conclusions:**

Results from this study support the traditional use of the selected medicinal plants in the management of arthritis and other inflammatory conditions. The free radical scavenging capacity of the extracts may be related to an immune boosting potential.

## Background

Free radicals or reactive oxygen species (ROS) are highly unstable molecules produced by living organisms during normal cellular metabolism [1]. A shift in the balance between oxidants and antioxidants in favour of oxidants is termed “oxidative stress” [2]. Oxidative stress is the leading predisposing factor of diseases such as cancer, diabetes, arthritis, rheumatoid arthritis, neurodegenerative disorders, hypertension, atherosclerosis and chronic inflammatory disorders.

Reactive oxygen species act by oxidising polyunsaturated fatty acids within cell membranes and lipoproteins via metal ion-dependent hydroxyl radical formation causing disruption of the cell membrane [3]. Proteins exposed to free radical attack may fragment or aggregate, adversely interfering with ion channels, cell receptors and oxidative phosphorylation [4].

The antioxidant constituents of medicinal plants may contribute to the protection of humans and animals from a variety of metabolic and infectious diseases [2, 5]. The intake of natural antioxidants has been inversely associated with morbidity and mortality from degenerative disorders and other infections [6]. The determination of antioxidant capacity is reaction-mechanism dependent and closely linked to the complex nature of phytochemicals [7].

Clinical symptoms observed in tissue wounds and other infectious diseases have been documented by humans since antiquity [8]. Inflammation is the organism’s response to the presence of pathogens, foreign body lodgement in tissues or injurious chemicals. The activation of inflammatory cells, among them monocytes and macrophages, which play an important role in innate immunity is an early indicator of this process. These cells are responsible for the appearance of the specific markers of inflammation such as oedema, heat, redness, pain and loss of function [8, 9]. Mediators of inflammation are chemical substances released from injured or activated macrophages that coordinate inflammatory responses. Examples are nitric oxide (NO), leukotrienes, lipoxins and prostaglandins [10].

The enzymatic action of nitric oxide synthase (NOS) on L-arginine results in the production of NO. There are two endothelial forms of NOS: Constitutive NOS (cNOS) and inducible NOS (iNOS). During inflammation, the amount of NO produced by iNOS is greater than that produced by cNOS [11]. Nitrite and nitrate are stable metabolites of NO used as molecular markers to indirectly determine the quantity of NO produced by the cells [12]. Elevated nitric oxide synthesis after the induced expression of NOS-2 by activated macrophages, is one of the main cytostatic, cytotoxic and pro-apoptotic mechanisms participating in the innate response in many mammals [12, 13]. Evans and Stefanovic-Racic [14] reported that osteoarthritic and rheumatoid joints produce NO locally, and inhibitors of NO synthases have strong anti-inflammatory activity.

Lipoxygenases (LOXs) are a group of oxidative enzymes with a non-heme iron atom in their active site. They are involved in the regulation of inflammatory responses by the generation of pro-inflammatory mediators known as leukotrienes or anti-inflammatory mediators known as lipoxins [15]. In general, lipoxygenases are classified as 5–, 8–, 12–, or 15-lipoxygenases according to their selectivity in oxygenating fatty acids in a specific position [16]. These enzymes catalyse the insertion of oxygen into poly-unsaturated fatty acids (PUFAs) such as arachidonic acid and linoleic acid. Lipoxygenases catalyse the formation of hydroperoxyeicosatetraenoic acids (HPETEs) from arachidonic acid. These HPETEs are subsequently reduced and transformed to form eicosanoids, which are involved in the development of rheumatoid arthritis, psoriasis, asthmatic responses and glomerulonephritis [15, 17].

Arthritis is a common problem observed in elderly people. Nearly one-fifth of the world’s population suffers from this debilitating disease [18]. The management of arthritis and other inflammatory disorders involves the use of different classes of drugs such as non-steroidal anti-inflammatory drugs (NSAIDs), corticosteroids and disease modifying anti-rheumatic drugs (DMARDs). The use of NSAIDs has gastrointestinal side effects, which includes irritation of the gastric mucosa, belching, gastric ulceration and bleeding. Long-term use of NSAIDs may impair renal and hepatic functions, predisposing the patient to cardiovascular diseases [19]. Hence, there is a continuous search for alternative drugs from plants and other natural sources.

Plants are excellent sources of antioxidants, anti-arthritic and anti-inflammatory agents [20, 21]. Pharmacological properties of plant extracts are attributed to the presence of phenols, flavonoids, tannins, flavonols, proanthocyanidins, nitrogenous compounds, vitamins and terpenoids [5, 22].

The nine South African medicinal plants used in this study were selected from the Phytomedicine database of the University of Pretoria based on traditional use in the treatment of arthritis, rheumatoid arthritis and other forms of inflammatory disorders [23]. Hence, this study aimed to investigate the validity of their traditional uses.

## Methods

### Chemicals and reagents

The chemicals we purchased from different suppliers were: Linoleic acid (Merck, Darmstadt, Germany), xylenol orange, ferric chloride (Searle Company, England), foetal calf serum (FCS), penicillin/streptomycin/fungizone (PSF) and Dulbecco’s modified Eagle’s medium (DMEM) (Highveld Biological, South Africa). Dulbecco’s phosphate buffered saline (PBS) (Lonza, Belgium), trypsin (Whitehead Scientific, South Africa). Quercetin, 3-(4,5- dimethylthiazol-2-yl)-2,5-diphenyl-tetrazolium bromide (MTT), sodium dodecyl sulphate, sodium nitrite, ferrous sulfate, and 15-lipoxygenase (Glycine max) (Sigma, Germany) and Tris(hydroxymethyl)aminomethane (Sigma, Switzerland), diclofenac sodium, Bovine Serum Albumin (BSA) (Sigma, St Louis USA). Sodium carbonate (Holpro Analytic, South Africa) 2,2-azino-bis (3-ethylben- zothiazoline-6-sulfonic acid) diammonium salt (ABTS), 2,2-diphenyl-1-picrylhydrazyl (DPPH), Folin–Ciocalteu reagent, gallic acid, 2,5,7,8-tetramethylchroman carboxylic acid (Trolox), potassium persulfate, glacial acetic acid, aluminium chloride, ascorbic acid, hydrochloric acid, dimethyl sulfoxide (DMSO), methanol and ethanol (Sigma-Aldrich St. Louis, MO, USA). Potassium ferric cyanide and iron (II) sulphate (Sigma, Germany).

### Collection of plant material

The leaves of nine different South African medicinal plants were collected from three locations namely the University of Pretoria Botanical Garden, Pretoria National Botanical Garden and Lowveld National Botanical Garden in the summer of 2013. Voucher specimens were prepared and deposited in the HGWJ Schweickerdt Herbarium of the University of Pretoria. The plant species with family and voucher specimen number in brackets were: *Hypericum roeperianum* G.W. Schimp.ex A. Rich. var. *roeperianum,* (Hypericaceae, PRU 120126)*, Cremaspora triflora* (Thonn.) K. Schum (Rubiaceae, PRU 120129)*, Heteromorpha arborescens* (Spreng.) Chan. & Schltdl (Apiaceae, PRU 120026)*, Pittosporum viridiflorum* Sims (Pittosporaceae, PRU 120025)*, Bolusanthus speciosus* (H. Bolus) Harms (Fabaceae, PRU 120027)*, Calpurnia aurea* (Aiton) Benth ssp aurea (Fabaceae, PRU 120125)*, Maesa lanceolata* Forssk (Maesaceae PRU120125)*, Elaeodendron croceum* (Thunb.) DC (Celastraceae, PRU 120127) and *Morus mesozygia* Stapf ex A. Chev (Moraceae, PRU 120128).

### Extraction

Acetone (technical grade, Merck) was used as an extractant in the assays using a ratio of 1:10 of pulverised dried leaf material to extractant. Acetone is the best choice as an extractant mainly due to its ability to extract compounds of a wide range of polarities [24], its non-toxicity to bioassay systems [25] and ease of removal from extracts. Three grams (3.0 g) of each tree leaf sample were extracted with 30 ml acetone [26]. The resulting suspension was vigorously shaken in 50 ml polyester centrifuge tubes for 5 min and centrifuged at 4000 × g for 10 min (Hettich Centrifuge, Rotofix 32A, Labotec, Johannesburg, South Africa). The extraction was repeated two more times on the marc and supernatants were decanted into preweighed glass vials after filtering through Whatman No. 1 filter paper and concentrated to dryness under a stream of cold air. The dried extracts were stored at 5 °C in tightly stoppered glass vials until use.

### Assay of nitric oxide production and viability of LPS- activated RAW 264.7 macrophages

#### Cell culture

The RAW 264.7 macrophage cells obtained from the American Type Culture Collection (Rockville, MD, USA) were cultured in a plastic culture flask in DMEM containing L-glutamine supplemented with 10 % FCS and 1 % PSF solution under 5 % CO_2_ at 37 °C. Cells were seeded in 96 well microtitre plates and were activated by incubation in medium containing LPS (5 μg/ml) alone (control) or LPS with different concentrations (100, 30, 10 and 2 μg/ml) of the extracts dissolved in DMSO. Quercetin served as a positive control NO inhibitor for the reduction of NO production [26].

#### Measurement of nitrite

Nitric oxide released from macrophages was determined by measuring the nitrite concentration in culture supernatant using the Griess reagent. After 24-h incubation, 100 μl of supernatant from each well of cell culture plates was transferred into 96-well microtitre plates and an equal volume of Griess reagent was added. The absorbance of the resultant solutions was determined on a BioTek Synergy microplate reader after 10 min at 550 nm. The concentrations of nitrite were derived from regression analysis using serial dilutions of sodium nitrite as a standard. Percentage inhibition was then calculated based on the ability of compounds to inhibit nitric oxide formation by cells compared with the control (cells in media without extracts), which was considered as 0 % inhibition.

#### Determination of cell viability

To determine whether the observed nitric oxide inhibition was not due to cytotoxicity, cytotoxicity was determined on the culture as described by Mosmann [27], with slight modifications. After removal of media, the cells were topped up with 200 μl DMEM. To each well, 30 μl of 15 mg/ml MTT was added. The cells were incubated at 37 °C in 5 % CO_2_. After 2 h, the medium was carefully removed and discarded and the formed formazan salt was dissolved in DMSO. The absorbance was read at 570 nm on a BioTek Synergy microplate reader. The percentage cell viability was calculated with the control value (cells without extracts containing LPS) taken as 100 % viability.

#### Determination of 15-lipoxygenase inhibitory assay

The assay was performed according to the method of Pinto et al. [28] with slight modifications. The assay is based on measuring the formation of the complex Fe3+/xylenol orange in a spectrophotometer at 560 nm. Lipoxygenase from *Glycine max* was incubated with 25 μl extracts (final concentration 3.6–454.5 μg/ml) or a standard inhibitor (Quercetin, final concentration 0.36–45.5 μg/ml) at 25 °C for 5 min. Then linoleic acid (final concentration, 140 mM) in borate buffer (50 mM, pH 7.4) was added and the mixture was incubated at 25 °C for 20 min in the dark. The assay was terminated by the addition of 100 μl of FOX reagent consisting of sulphuric acid (30 mM), xylenol orange (100 mM), iron (II) sulfate (100 mM) in methanol/water (9:1). For the negative control, only LOX solution and buffer were pipetted into the wells. Blanks (background) contained the enzyme LOX during incubation, the substrate (linoleic acid) was added to the FOX reagent. The lipoxygenase inhibitory activity was determined by calculating the percentage of inhibition of hydroperoxide production from the changes in absorbance values at 560 nm after 30 min at 25 °C.

$$ \%\ \mathrm{inhibition} = \left[\left({\mathrm{A}}_{\mathrm{control}}\hbox{--} {\mathrm{A}}_{\mathrm{b}\mathrm{lank}}\right)-\left({\mathrm{A}}_{\mathrm{sample}}\hbox{--} {\mathrm{A}}_{\mathrm{b}\mathrm{lank}}\right)/\left({\mathrm{A}}_{\mathrm{control}}\hbox{--} \mathrm{A}{\mathrm{b}}_{\mathrm{lank}}\right)\right]\times 100. $$

Where, A_control_ is the absorbance of control well, A_blank_ is the absorbance of blank well and A_sample_ is the absorbance of the sample well.

#### Anti-arthritic (protein denaturation assay)

Anti-arthritic activity of the extracts was determined using the method of Sakat et al. [29] with minor modifications. The reaction mixture consisted of the 100 μl test extracts (final concentration 9.77–1250 μg/ml) and 100 μl of 5 % aqueous solution of bovine serum albumin (BSA); pH was adjusted adding a small volume of glacial acetic acid. The sample extracts were incubated at 37 °C for 20 min and then heated to 70 °C for 10 min. The mixture was allowed to cool for 10 min after which turbidity was measured at 660 nm. The blank comprised the sample and distilled water. Distilled water was used as the negative control. The positive control was diclofenac sodium (final concentration 0.61–78. Percentage inhibition was calculated using the formula:

$$ \%\ \mathrm{inhibition} = 100*\left(\mathrm{Abs}\ \mathrm{Sample}\hbox{-} \mathrm{Blank}/\mathrm{control}\hbox{-} 1\right) $$

The IC_50_ was calculated from a graph of inhibition against the different concentrations. The experiment was carried out in triplicate.

#### The 2,2-azino-bis (3-ethylben- zothiazoline-6-sulfonic acid) diammonium salt (ABTS) antioxidant assay

The quantitative ABTS radical scavenging capacity of the extracts was measured using the 96 well microtitre plate method described by Re et al. [30]. Trolox and ascorbic acid were used as positive controls, methanol as the negative control and extracts without ABTS as blank. The absorbance was read at 734 nm exactly after 5 min of mixing. The percentage of ABTS^• +^ inhibition was calculated using the formula:

Scavenging capacity (%) = 100 - [(absorbance of the sample - absorbance of the sample blank) × 100/ (absorbance of control) – (absorbance of control blank)].

The IC_50_ values were calculated from the graph plotted as inhibition percentage against the concentration. A Trolox standard curve was drawn by plotting percentage inhibition of the ABTS^+^ radical against the concentration of Trolox. Data from the test extracts were analysed in a similar manner and the gradient obtained was divided by the gradient of the Trolox reaction to give a Trolox equivalent antioxidant capacity (TEAC) value.

#### The 2,2-diphenyl-1-picrylhydrazyl (DPPH) antioxidant scavenging assay

The DPPH radical scavenging activity of acetone extracts was determined using the method described by Brand-William et al. [31] based on the reduction of DPPH in the presence of a hydrogen-donating antioxidant in 96-well microtitre plates. One hundred and sixty microlitres of the methanolic solution of DPPH (0.04 mg/ml,) was added to 40 μl ascorbic acid and Trolox at concentrations of 1.0–200 μg/ml (positive controls), and different concentrations of crude extracts (3.9–500 μg/ml). After 30 min, the absorbance was measured at 517 nm using a Biotek microplate reader. The analysis was carried out in triplicate, and the results were expressed as the percentage reduction of the initial DPPH absorption in relation to the control group. The concentration of extract that reduced DPPH colour by 50 % (IC_50_) was determined using the formula in the previous paragraph.

#### Ferric reducing antioxidant power (FRAP) assay

The FRAP of the nine acetone leaf extracts was determined by direct reduction of potassium ferricyanide (K_3_Fe (CN)_6_) to potassium ferrocyanide (K_4_Fe(CN)_6_) (electron transfer process from the antioxidant). The increase in absorbance from the formation of Pearl’s Prussian blue complex following the addition of excess ferric ion was measured as described by Berker et al. [32] with some modifications. The reaction medium (210 μl) containing 40 μl of the test samples or positive controls (Trolox and ascorbic acid; concentration range between: 15.62–2000 μg/ml); 100 μl of 1.0 M hydrochloric acid; 20 μl of 1 % (w/v) of SDS; 30 μl of 1 % (w/v) of potassium ferricyanide, was incubated for 20 min at 50 °C, then cooled to room temperature. Finally, 20 μl of 0.1 % (w/v) of ferric chloride was added. The absorbance was read at 750 nm. The blank absorbance was prepared as above without the addition of ferric chloride. The TEAC (Trolox Equivalent Antioxidant capacity) was calculated by dividing the slope of each sample (slope obtained from the line of best fit of the absorbance against concentration using the linear regression curve) by that of Trolox.

#### Total phenolic content (TPC) determination

The total phenolic content (TPC) was determined colorimetrically using a Folin-Ciocalteu 96-well microplate assay developed by Zhang et al. [33]. The total phenolic content was calculated from the linear equation of a standard curve prepared with gallic acid and expressed as Gallic Acid Equivalent (GAE) per g of extract.

#### Total flavonoid content (TFC) determination

Total flavonoid content was determined using the method of Ordonez et al. [34]. A volume of 0.5 ml of 2 % AlCl_3_ in ethanol solution was added to 0.5 ml of sample solution (1 mg/ml). After one hour at room temperature, the absorbance was measured at 430 nm. A yellow colour is indicative of the presence of flavonoids. Total flavonoid content was calculated and expressed as mg quercetin equivalent/g of crude extract using a standard curve prepared with quercetin.

#### Statistical analysis

All experiments were conducted in triplicate and values expressed as the mean ± standard deviation. Variations in mean were calculated using one-way Analysis of variance (ANOVA), and means were statistically significant if *p <*0.05. Post-Hoc analyses were carried out using Tukey HSD or Dunnett Multiple comparison tests on SPSS V.23 (Social Program for Social Sciences, SPSS Corporation, Chicago, IL).

## Results and Discussion

### Antioxidant activities, total phenolic and flavonoid contents of the tested extracts

DPPH is a stable organic free radical with an absorption band at 517 nm. It loses the purple colour that absorbs at this wavelength when accepting an electron or a free radical species, which results in a yellow colour [35]. The extracts had varying degrees of antioxidant activity in the DPPH assay. The IC_50_ values ranged between 7.72 and 154.77 μg/ml. Acetone leaf extracts of *E. croceum*, *M. lanceolata* and *H. roeperianum* had good radical scavenging activity with IC_50_ values of 7.72, 12.95 and 34.04 μg/ml respectively (Table 1). The IC_50_ value of a compound is inversely related to its antioxidant capacity. A lower IC_50_ value indicates a stronger antioxidant activity of the extract or compound [36]. Ascorbic acid, a known potent antioxidant, had the highest DPPH scavenging activity (3.30 ± 0.06 μg/ml). *E. croceum* with the highest level of total phenolics had the lowest IC_50_ value, higher than that of ascorbic acid but close to Trolox (Table 1). *Elaeodendron croceum* had the highest trolox equivalent antioxidant capacity (TEAC) value (1.32), there was no statistical difference when the IC_50_ mean of *E. croceum* extracts was compared with trolox (*p >* 0.05). *Calpurnia aurea* had the weakest antioxidant capacity (0.06) in this assay (Table 1). The IC_50_ values for ABTS assay ranged from 3.05 to 96.47 μg /ml. Antioxidant activity of *E. croceum* acetone extracts compared well with Trolox, *p >* 0.05 (Table 1). The trend for the ferric reducing capacity of the extracts was similar to those of the DPPH and TEAC. *E. croceum* showed the highest reducing power (0.99) (Table 1).

**Table 1:** Results of the quantitative phytochemical content analysis and antioxidant activity of the acetone leaf extracts of the nine selected plants

Plant species	TPC (GAE mg/g)	TFC (QE mg/g)	DPPH IC_50_ (μg/mL)	ABTS IC_50_ (μg/mL)	TEAC	FRAP
*Hypericum roeperianum*	472.40 ± 1.15^a^	515.20 ± 1.53^a^	34.04 ± 0.25^a^	27.93 ± 2.98^a^	0.15 ± 0.02^a^	0.23 ± 0.03^a^
*Cremaspora triflora*	296.07 ± 5.86^b^	186.53 ± 2.65^b^	85.48 ± 2.17^b^	45.77 ± 1.67^b^	0.13 ± 0.02^a^	0.16 ± 0.02^a^
*Heteromorpha arborescens*	255.07 ± 5.03^c^	275.53 ± 4.58^c^	154.77 ± 4.07^c^	95.67 ± 2.91^c^	0.07 ± 0.01^a^	0.06 ± 0.00^a^
*Pittosporum viridiflorum*	423.40 ± 1.15^d^	389.20 ± 3.21^d^	50.29 ± 1.23^d^	58.40 ± 2.68^d^	0.09 ± 0.01^a^	0.25 ± 0.07^a^
*Bolusanthus speciosus*	377.73 ± 2.65^e^	726.20 ± 3.79^e^	115.12 ± 4.81^e^	59.96 ± 4.23^d^	0.10 ± 0.02^a^	0.07 ± 0.01^a^
*Calpurnia aurea*	308.07 ± 2.89^f^	285.20 ± 8.50^c^	170.40 ± 4.16^f^	96.47 ± 8.09^e^	0.06 ± 0.01^a^	0.07 ± 0.01^a^
*Maesa lanceolata*	669.07 ± 3.79^g^	223.53 ± 7.55^f^	12.95 ± 0.78^g^	9.64 ± 0.45^f^	0.70 ± 0.08^b^	0.15 ± 0.01^a^
*Elaeodendron croceum*	958.40 ± 1.53^h^	273.86 ± 5.03^c^	7.72 ± 0.27^g,k^	3.05 ± 0.27^f^	1.32 ± 0.17^c^	0.99 ± 0.08^b^
*Morus mesozygia*	375.07 ± 3.51^e^	657.53 ± 2.65^g^	106.98 ± 2.42^h^	45.5 ± 3.22^b^	0.14 ± 0.01^a^	0.18 ± 0.03^a^
Trolox	NA	NA	5.58 ± 0.05^g,k^	6.82 ± 0.99^f^	1.00 ± 0.00^b,c^	1 ± 0.00^b^
Ascorbic acid	NA	NA	3.30 ± 0.06^k^	2.92 ± 0.21^f^	2.32 ± 0.28^d^	3.67 ± 0.42^c^

Values with different letters are significantly different at *p <* 0.05; NA = Not Applicable

Phenolics exert their antioxidant effects by decreasing oxygen concentration, intercepting singlet oxygen, preventing first chain initiation by scavenging initial radicals, such as hydroxyl radicals, binding metal ion catalysts, decomposing primary products of oxidation to non-radical species and breaking chains to prevent continued hydrogen abstraction from substances [36, 37].

The total phenolic content of the acetone extracts ranged from 255.07 to 958.40 mg GAE/g. *E. croceum*, *M. lanceolata* and *H. roeperianum*, had the highest amount of TPC, with values of 958.40, 669.07 and 472.40 mg gallic acid equivalent (GAE)/g respectively (Table 1). There is a strong negative correlation when the antioxidant activities of the extracts were compared to their TPC values. For DPPH (r = − 0.79, *p <* 0.05), ABTS (r = − 0.82, *p <* 0.05) indicating that the higher the total phenolic content of the acetone extracts the lower their IC_50_ values and therefore the highest the antioxidant activity. There was a strong positive correlation between TPC and TEAC (r = 0.96, *p <* 0.05) and FRAP (r = 0.87, *p >* 0.05) values (Table 1). *E. croceum* had a higher antioxidant capacity (1.32 ± 0.17) than trolox, and a value of 0.99 ± 0.08 in the FRAP analysis. The high TPC values of *E. croceum*, *M. lanceolata* and *H. roeperianum* extracts are probably responsible for their excellent antioxidant activities (Table 1).

The total flavonoid content (TFC) values ranged from 186.53 to 726.20 mg QE/g. *Bolusanthus speciosus*, *Morus mesozygia* and *Hypericum roeperianum* had the highest TFC (Table 1). There was a poor correlation between TFC and antioxidant activity. For DPPH (r = 0.15, *p >* 0.05), ABTS (r = 0.04, *p >* 0.05), TEAC (r = −0.34, *p >* 0.05) and FRAP (r = −0.20, *p >* 0.05) indicating that the antioxidant capacity of the extracts is not caused by total flavonoid content (Table 1).

There were no statistically significant differences in mean of the results obtained via DPPH and ABTS assays (*p >* 0.05). This may imply that any one of the methods would have given a representative value of the antioxidant activity of the extracts. The results were comparable, probably due to a similarity in the mechanism of scavenging activities of the extracts [38].

### 15-lipoxygenase inhibitory activity

**Figure 1:**
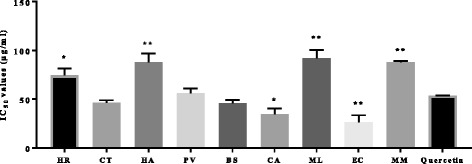
The anti-lipoxygenase activity of the acetone leaf extracts of the different plant species, showing the IC_50_ value of the extracts and quercetin. * = Indicates significant difference compared to quercetin (*p <* 0.05), ** = Indicates significant difference compared to Quercetin(*p <* 0.01). HR = *Hypericum roeperianum*, CT = *Cremaspora triflora*, HA = *Heteromorpha arborescens*, PV = *Pittosporum viridiflorum*, BS = *Bolusanthus speciosus,* CA = *Calpurnia aurea*, ML = *Maesa lanceolata*, EC = *Elaeodendron croceum*, MM = *Morus mesozygia*

Lipoxygenase enzymes in the cells of the body catalyse the conversion of arachidonic acid to hydroperoxyeicosatetraenoic acids (HPETEs), which are then reduced to mono-HETEs or diHETEs and leukotrienes; these are ranked amongst the most potent natural mediators of hypersensitivity and inflammation [39]. A great number of plant-derived constituents exhibit a pleiotropic spectrum of anti-inflammatory actions [9]. The ferrous oxidation-xylenol orange (FOX) assay was used to determine the 15-lipoxygenase inhibitory activity of the crude extracts. The results presented in Figure 1 indicate that all the extracts have varying degrees of inhibitory activity against 15-lipoxygenase, thus supporting claims of their use in the management of arthritis, aches, pains, infections, rheumatism and other inflammatory disorders [23]. The IC_50_ values of the extracts ranged from 26.23 to 91.77 μg/ml respectively. *E. croceum* and *C. aurea* acetone crude extracts had very good activity against the 15-lipoxygenase enzyme, with IC_50_ values of 26.23 and 34.70 μg/ml respectively. These extracts compared better than the positive control quercetin which had IC_50_ value of 53.69 μg/ml. *Bolusanthus speciosus* and *Cremaspora triflora* also had promising inhibitory anti-inflammatory activities, with both IC_50_ values < 50 μg/ml (Figure 1). The observed variation in the strength of inhibition of 15-lipoxygenase activity may be attributed to differences in the phytochemical composition of the various extracts [9].

Generally, there was a strong, inverse correlation between the anti-lipoxygenase activity of *E. croceum*, *C. aurea*, *B. speciosus*, and *C. triflora* and the phenolic contents (r = −0.80, *p <* 0.05) of these extracts. The promising anti-lipoxygenase activity of the acetone leaf extracts of *B. speciosus* may be linked to the high concentration of flavonoids (Table 1 and Figure 1). Bojase et al. [40] showed the presence of flavonoids in the stem bark of *B. speciosus*. Flavonoids are known to interfere with the different stages of the arachidonate cascade via cyclooxygenase or lipoxygenase pathways to alleviate inflammatory responses [41].

### Nitric oxide production and viability of LPS-activated RAW 264.7 Macrophages

Nitric oxide (NO) has long been recognised as an important molecule involved simultaneously in the regulation of apoptotic death and cell viability [10]. Activation of RAW 264.7 macrophages with lipopolysaccharide (LPS) induces the production of NO. The extent of NO production is determined by measuring the concentration of nitrite, a stable oxidised product of NO [42]. All extracts had a concentration-dependent inhibition of NO production (Table 2). The good NO inhibitory activity of *M. lanceolata* and *B. speciosus* at a concentration of 100 μg/ml is probably due to their toxicity to macrophages with percentage cell viabilities of 9.29 % and 34.25 % respectively (Table 2). The extracts of *Heteromorpha arborescens* and *Hypericum roeperianum* at 30 μg/ml and 10 μg/ml had significant NO inhibitory activity. In addition, extracts of *M. lanceolata* inhibited NO production by 47.53 % at the lowest concentration (2 μg/ml). The capacity of the extracts to inhibit NO production may be attributed to their phenolic contents. Phenols regulate the synthesis of inducible nitric oxide synthase (iNOS) by inhibiting the nuclear transcription factor NFkappaB [43]. Many natural compounds from medicinal plants inhibit the expression of iNOS in LPS-activated macrophages. For example, phenolics isolated from *Yucca schidigera* have antioxidant and free-radical scavenging capacity, which aid in suppressing reactive oxygen species that stimulate inflammatory response [43].

**Table 2:** Inhibitory activities of the nine South African medicinal plants used traditionally in the management of different inflammatory disorders on nitric oxide production and cell viability in LPS-activated RAW 264.7 macrophages

Plants	Concentration (μg/mL)	NO (μM)	% NO inhibition	% Macrophage viability
*Hypericum roeperianum*	100	2.46 ± 0.17	89.72	50.40
	30	4.55 ± 0.25	81.04	89.40
	10	8.28 ± 0.17	65.90	97.53
	2	16.93 ± 1.43	29.41	100
*Cremaspora triflora*	100	6.15 ± 0.34	74.34	88.22
	30	16.21 ± 0.25	32.39	89.71
	10	18.36 ± 0.76	23.45	97.58
	2	20.32 ± 1.52	15.26	100
*Heteromorpha arborescens*	100	0.08 ± 1.52	99.65	45.13
	30	2.52 ± 0.08	89.48	82.12
	10	7.52 ± 0.59	68.63	95.90
	2	23.77 ± 0.17	0.87	100
*Pittosporum viridiflorum*	100	0.32 ± 0.17	98.66	95.66
	30	4.67 ± 0.08	80.54	100
	10	16.69 ± 0.25	30.40	100
	2	24.43 ± 0.25	1.86	100
*Bolusanthus speciosus*	100	0.44 ± 0.84	98.16	34.25
	30	9.25 ± 0.17	61.43	100
	10	15.32 ± 0.17	36.11	98.35
	2	19.55 ± 0.08	18.49	99.80
*Calpurnia aurea*	100	1.21 ± 0.25	94.94	60.27
	30	18.60 ± 0.08	22.46	100
	10	21.04 ± 0.00	12.29	96.50
	2	23.30 ± 0.17	2.85	79.51
*Maesa lanceolata*	100	0.01 ± 0.00	100	9.29
	30	0.20 ± 0.00	99.16	16.96
	10	10.44 ± 0.17	56.47	22.12
	2	12.58 ± 0.17	47.53	69.63
*Elaeodendron croceum*	100	1.21 ± 0.25	94.94	48.92
	30	6.93 ± 0.25	71.11	69.65
	10	14.31 ± 0.08	40.33	90.22
	2	20.74 ± 0.42	13.53	>100
*Morus mesozygia*	100	0.20 ± 0.12	99.16	57.07
	30	11.15 ± 0.12	53.49	100
	10	20.68 ± 0.36	13.77	100
	2	23.18 ± 0.12	3.35	95.05
Quercetin	100	0.79 ± 0.00	83.12	37.71
	30	1.10 ± 0.00	100	51.89
	10	1.40 ± 0.00	100	68.79
	2	1.44 ± 0.00	100	66.30

### In vitro anti-arthritic activity

**Figure 2:**
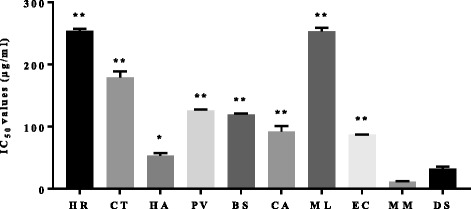
The protein anti-denaturation activity of acetone leaf extracts of different plant species, showing the IC_50_ values of the extracts and Diclofenac sodium. * = indicates significant difference in mean, when the diclofenac sodium was compared to the extracts(*p <* 0.05), ** = indicates significant difference in mean of extract compared to diclofenac sodium, (*p <* 0.01). HR = *Hypericum roeperianum*, CT = *Cremaspora triflora*, HA = *Heteromorpha arborescens*, PV = *Pittosporum viridiflorum*, BS = *Bolusanthus speciosus,* CA = *Calpurnia aurea*, ML = *Maesa lanceolata*, EC = *Elaeodendron croceum*, MM = *Morus mesozygia,* DS = Diclofenac sodium

The anti-denaturation study for investigating anti-arthritis activity was performed using bovine serum albumin (BSA). When BSA is heated, it undergoes denaturation and antigens are expressed which are associated with type-III hypersensitivity reaction, which in turn is related to diseases such as serum sickness, glomerulonephritis, rheumatoid arthritis and systemic lupus erythematosus [44]. All the extracts had a dose-dependent response in the in vitro anti-arthritic test. The IC_50_ ranged from 11.89 to 254 μg/ml. *M. mesozygia* and *H. arborescens* had good anti-denaturation activity (Figure 2), with IC_50_ values of 11.89 and 53.74 μg/ml respectively. *M. mesozygia* extracts were three times more effective than the positive control, diclofenac sodium (IC_50_ = 32.37 μg/ml). The promising activities of the extracts support the traditional claims of use as remedies for arthritis, rheumatism and other chronic inflammatory conditions [23]. Denaturation of protein is one of the causes of rheumatoid arthritis. Production of autoantigen in certain arthritic diseases may be due to denaturation of protein. The mechanism of denaturation probably involves alteration of electrostatic, hydrogen, hydrophobic and disulphide bonds [45].

## Conclusions

The in vitro study of the acetone leaf extracts of the nine selected plants revealed promising antioxidant, anti-inflammatory and anti-arthritic activity. The study corroborates traditional claims of the use of these South Africans medicinal plants in the management of arthritis, infections, rheumatism and inflammation. The plants have potential for development as therapeutic agents of inflammation and other autoimmune disorders, but safety will have to be examined in more detail. Since the mechanism of action of these extracts has not been elucidated, it is recommended to investigate this aspect as part of the on-going drug development process. It may be interesting to correlate the activities with the chemical composition of the different plant extracts to determine if other correlations exist.
